# The Open-Air Treatment of Tuberculosis

**Published:** 1898-08-06

**Authors:** 


### EDITOR'S LETTER-BOX.

[Our Correspondents are reminded that prolixity is a great bar to publication, and that brevity of style and conciseness of statement greatly facilitate early insertion.]

THE OPEN-AIR TREATMENT OF TUBERCULOSIS.

Referring to an article in The Hospital, for July 30th, on this subject, Dr. R. W. Philip, of Edinburgh, writes to correct a misapprehension which he thinks it might give rise to in regard to the treatment of the patients at the Victoria Hospital. He says: "The patients in the Victoria Hospital live during the greater part of the day out of doors, even in wet weather, and my contention is that the results have been so good that I see no reason why the Open-Air treatment should not be universally applied in all parts of the United Kingdom with success." We should be sorry if any of our readers should have been led by our remarks to think lightly of the efforts made by Dr. Philip to secure for his patients the advantages of the Open-Air treatment. He is well known as having been a pioneer in the matter at a time when this line of treatment had not received the recognition which has lately been accorded to it; and if the article in The Hospital had been directed to a consideration of the treatment at the Victoria Hospital, we should have given a full description of the arrangements there made for carrying it out. But that was not the object of the article to which Dr. Philip refers. We wished to point out the desirability of restricting the term Open-Air treatment to treatment of a certain character, and the very fact that the paragraph which we quoted, containing as it did details as to the temperature at which the wards were maintained, and as to the arrangement of the window sashes, should have occurred in the course of a description of this treatment, seemed to us, and still seems to us, to clinch the importance of the warning which we gave.

